# Presence of necrotic strains of *Potato virus Y *in Mexican potatoes

**DOI:** 10.1186/1743-422X-6-48

**Published:** 2009-05-06

**Authors:** Victoriano Roberto Ramírez-Rodríguez, Katia Aviña-Padilla, Gustavo Frías-Treviño, Laura Silva-Rosales, Juan Pablo Martínez-Soriano

**Affiliations:** 1 Centro de Investigación y de Estudios Avanzados del Instituto Politécnico Nacional, Campus Guanajuato, km. 9.6 libramiento norte, carretera Irapuato-León, 36821 Irapuato, Guanajuato, Mexico; 2 Universidad Autónoma Agraria Antonio Narro, Departamento de Parasitología, Buenavista, Saltillo, Coahuila, Mexico

## Abstract

As part of a routine screening for the possible presence of the necrotic strains of potato virus Y affecting potatoes in Mexico, five PVY isolates were submitted to biological and molecular analysis. Considering their serological properties, two belong to the common strain (O) and three to the necrotic strain (N). All the isolates induced vein necrosis in *Nicotiana tabacum*. To characterize the isolates, 5' NTR and P1 genes were sequenced and compared with sequences from GenBank database. Nucleotide sequence similarity ranged from 47–100% in the 5' NTR and from 63–100% in the P1 coding region. The lowest amino acid similarity between sequences of P1 gene was 55%. In phylogenetic trees of 5'NTR analysis, two PVY^O ^Mexican isolates clustered with other PVY^O ^isolates. In turn, the three PVY^N ^isolates grouped with PVY^N-NTN ^isolates. The phylogenetic analysis of P1 sequences (nucleotide and amino acid) showed two PVY^O ^isolates grouping next to N-NTN cluster. A detailed analysis of the PVY^O ^isolates showed two potential recombination regions in the P1 gene, in contrast to 5'NTR where no recombination was detected.

## Background

Potato virus Y (PVY), the type member of the family Potyviridae, can infect potato, tobacco, tomato and pepper as well as wild species, especially those in the *Solanaceae *family [1]. The conventional classification of PVY isolates is based on primary hosts, symptoms induced in differential plants and serological reaction to monoclonal antibodies. The isolates reported so far, have been classified in three main strains: PVY^N^, PVY^O ^and PVY^C ^[2]. Isolates belonging to the PVY^N ^strain induce severe vein necrosis on *Nicotiana tabacum *leaves. This strain has been divided into two groups: the first one causing mild mosaic in most potato cultivars, while the second one provokes "potato tuber necrotic ring disease" (PTNRD) and severe chlorotic mosaic in the leaves [3]. It also produces veinal necrosis in tobaco leaves and is referred as PVY^NTN ^[NTN = isolates belonging to the necrotic group (N) of PVY and inducing tuber necrosis (TN)], according to a decision of the European Association of Potato Research Virology Section [4,5]. The PVY^O ^strain isolates induce non-necrotic mosaics on tobacco leaves but more severe symptoms on potato, such as crinkling, leaf dropping or severe necrotic mosaic. The PVY^C ^strain causes stipple streak on potato cultivars carrying the Nc resistance gene and non-necrotic symptoms, similar to those of PVY^O^, on *N. tabacum *leaves [6]. The symptoms of mosaic are masked in temperatures out of the normal rank from 10°C to 25°C.

The serological classification of PVY isolates is a matter of discussion. Coat protein-directed polyclonal antibodies do not discriminate between PVY strains so monoclonal antibodies specific to O and N strains have been used to characterize selected PVY isolates [7,8]. Moreover, some isolates were determined as PVY^O ^using monoclonal antibodies, nevertheless induced tobacco vein necrosis, which are but infectious and induce less severe symptoms in potato than the other PVY^N ^isolates and it has been called PVY^N^-Wilga isolate [9,10]. Which shows that the serological and pathogenic traits of a determined PVY isolate seem not to have an absolute relationship and, on other hand, some serological detections have not showed the specificity expected [2,5,8,11].

Conventional methods of PVY classification do not result in a universal criterion for grouping virus isolates within species. Complete genomic nucleotide sequence analysis of isolates which showed that the degree of similarity differs across the genome, being the 5' terminal untranslatable segment the most variable region of the PVY genome [12]. This has led to a re-evaluation of the subgroup based on gene sequences analysis, which has led to an alignment with the phenotype-based classification with exceptions concerning the ability to induce tobacco veinal necrosis. The sequence-based clustering of all isolates reported so far. A comparative analysis of available sequences of necrotic and non necrotic isolates led to the hypothesis that the tobacco vein necrosis determinant is localized in the 3' terminal region covering the CP gene and 3' NTR [13]. Other studies using the CP and P1 genes and the 5' and 3' NTRs have concluded that those regions are not involved in the induction of vein necrosis in tobacco [14]. From de clustering and necrotizing properties, it has been suggested that the ability to cause vein necrosis in tobacco could be located in the 5' rather than in the 3' half of the viral RNA, in the HC-Pro protein specifically [15].

It has been suggested that the strain NTN of PVY resulted of the natural combination between PVY^O^, or PVY^C^, and PVY^N ^[16]. Isolates of PVY, which might be intermediate forms of the PVY^O ^and PVY^N ^groups, have been reported, sharing similar symptoms as well as serological and genomic properties with both groups [17]. Moreover, it has been indicated upon comparisons of the 5'NTR and P1 sequences of PVY^N ^and PVY^NTN ^from American and European origin, that they formed separate geographic groups whit a 98% or higher similarities between them [18]. This suggests that the NTN strain detected in some geographic region may have arisen from an N strain of the same region. This may explain the difference among NTN isolates of different geographic regions in the world. A similar finding is reported from amino acid sequence analyses of the capsid protein gene of American and Japanese isolates [11].

To strengthen the PVY group classification and to determine the relationships between Mexican isolates and PVY isolates from other parts of the world, we determined the nucleotide sequence of the 5'NTR and P1 coding region for ten Mexican isolates and their biological characterization with six plants species. This is the first report of PVY^W ^and PVY^N ^presence in Mexico.

## Results

### ELISA and biological tests

Two Mexican isolates showed positive reaction with PVY^O ^antibodies, Pic3 and Vic20 isolates (Table 1). The isolates were inoculated to six plants species (*Capsicum annuum *var. Serrano; *Chenopodium quinoa; Solanum lycopersicum*; *Nicotiana tabacum *cv. Burley; *Physalis floridana *and *S. tuberosum *cv. Alpha) (data no shown). In three species (*N. tabacum*, *Ph. floridana *and *S. tuberosum*) symptoms were more severe than in the other species tested. Three isolates belonging to the N strain, Pic1, Vic6 and Vic15 (Table 1), inoculated in plants of *N. tabacum *induced typical necrotic symptoms; i.e. vein necrosis 7–10 days after inoculation. The Pic3 and Vic20 isolates, both PVY^O ^(Table 1), induced severe chlorotic mosaics on tobacco leaves as initial symptoms (7–10 days after inoculation) and moderate vein necrosis plus severe distortion of leaves three weeks post-inoculation. The aggressiveness of PVY Pic3, Vic6, Vic15 and Vic20 isolates on *N. tabacum *as well as the severity of symptoms showed by *Ph. floridana *plants infected with PVY Pic3, Vic6, Vic15 and Vic20 isolates was remarkable.

**Table 1:** Serological reaction from five PVY Mexican isolates against eight antibodies, five of them for five different potato viruses and three for three PVY strains.

	Reaction to antibodies							
Isolate	PVA	PLRV	PVS	PVX	PVY	PVY^O+C^	PVY^O^	PVY^N^
Pic1	-	-	-	-	+	-	-	+
Pic3	-	-	-	-	+	-	+	-
Vic6	-	-	-	-	+	-	-	+
Vic15	-	-	-	-	+	-	-	+
Vic20	-	-	-	-	+	-	+	-

### Sequence analysis

#### 5'-NTR

Twentynine nucleotide sequences of PVY 5'NTR were analyzed: (five O, sixteen N and eight NTN strains respectively). Of these sequences five were from Mexico (this report, GenBank accession numbers AY700016–AY700020) and twentyfour from other parts of the world. The Mexican isolates of PVY^O^, namely Pic3 and Vic20 grouped in a cluster with those others identified as O and N strains (O-N cluster) while the PVY^N ^isolates (Vic6, Vic15 and Pic1) grouped in a different cluster with other N strains, besides all N-NTN strains in N-NTN cluster (data no shown). The group "N-NTN" shows particular clusters of the three Mexican PVY^N ^isolates, along with North American and European PVY^N-NTN ^isolates. On the other hand, the cluster "O-N" shows a specific grouping of two PVY^O ^isolates: PVY^O^-Pic3 with PVY^N^-Wi-P isolate (from Poland) and PVY^O^-Vic-20 with PVY^O^-PO7 isolate (from Canada). The twenty nine 5'NTR nucleotide analyzed ranged in similarity from 58 to 100%, and there were no recombination sites detected after testing with the RDP program (data not shown).

#### P1 gene

Thirty four sequences of the whole P1 gene (five from Mexico) were analyzed, where they are included to O strain (eight isolates), N strain (eighteen isolates) and NTN strain (eight isolates), yielding a percentage of similarity ranking between 63–100%. The general clustering shows two main groups: "O-N" and "N-NTN" (data not shown). In the group "N-NTN" there is a subcluster composed by three isolates: two Mexican PVY^O ^isolates (Pic3 and Vic20) with the PVY^N^-Wi-P Poland isolate (Accession Number AF248500), having the highest similarity among all the isolates analyzed: 96.5% (Vic20 and Wi-P) and 98.9% (Pic3 and Wi-P). In turn, the three Mexican isolates of the N strain showed a clustering with seven European isolates in the group "N-NTN" but separated from the cluster of nine North American isolates. The P1 gene amino acid sequences from the same isolates were analyzed; having a range in similarity of 55–100% and similar clustering to analysis of nucleotide sequences (data not shown).

**Figure 1:**
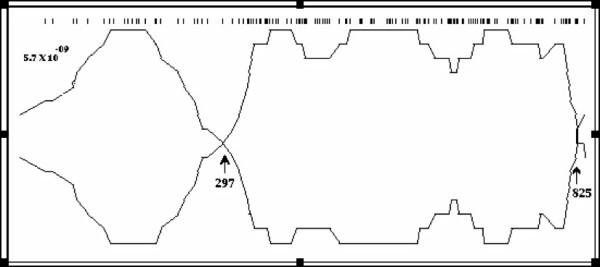
**Graphical representation of recombination detected in P1 gene nucleotide sequences from PVY^O^-Pic3 (Mexico) and PVY^N^-N 5yt (North America)**. Arrows indicate edges of potential recombination region. A window size of ten nucleotides and the maximum acceptable probability of 0.00001 were used.

The thirtyfour nucleotide sequences of the P1 gene were analyzed with the recombination program RDP. In this analysis, different windows sizes and different maximum acceptable probability values were used. In general, two potentially recombinant regions were detected in PVY^N^-N 5yt and two PVY^O ^Mexican isolates, detecting potential crossover sites in the nucleotides in position 297, 301, 321 and 825 (Figure 1).

**Figure 2:**
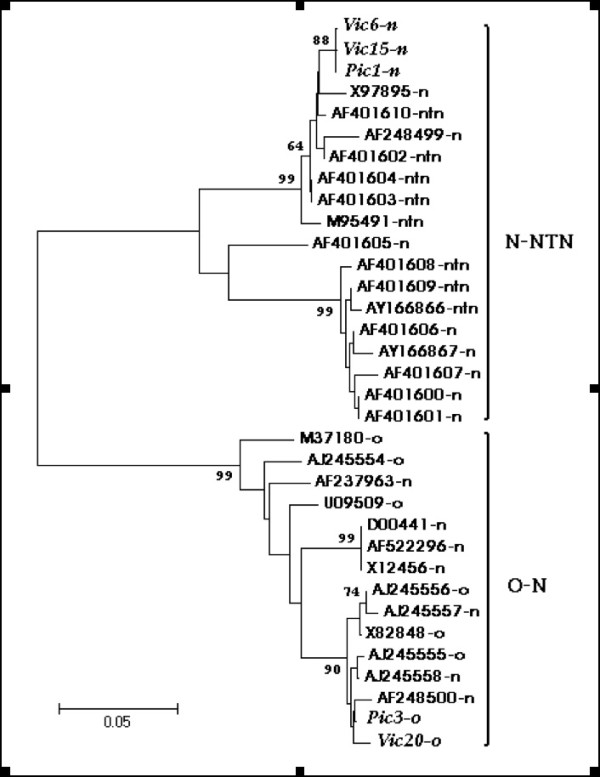
**Dendrogram of 34 nucleotide sequences (using 306 nucleotides from the 5' region of the P1 gene)**. The Pic3 and V20 isolates (PVY^O ^Wilga type both) cluster inside a "O-N" group. The Mexican isolates are in italics. Left to right: nucleotides from whole P1 gen of compared sequences (1 to 825 nt).

Based on the results obtained from the recombination analysis of P1 gene, the thirtyfour nucleotides sequences were analyzed in two regions. For this analysis the CLUSTALX, DNAStar, Mega3 and PAUP programs were used. One region that included the 519 nucleotides of the 3' end (with a similarity range of 62–100%), grouping the five Mexican isolates next to PVY^N-NTN ^isolates (data not shown). The other putative region for recombination included the 306 nucleotides near the 5' end (with a range of 64–100%), produced a dendrogram that showed two clusters: "O-N", where are included the Pic3, Vic20 and PVY^N^-Wi-P (Accession Number AF248500) isolates, and the "N-NTN" group which included Pic1, Vic6 and Vic15 Mexican isolates (Figure 2). The similarity between PVY^O ^isolates (Pic3 and Vic20) and PVY^N^Wi-P was 98.7–99.3%, the two Mexican PVY^O ^isolates have 66–67% similarity with three PVY necrotic isolates: Pic1, Vic6 and Vic15.

## Discussion

Nucleotide sequence analysis of two hundred sequences of different regions of PVY genome (data not shown) and thirteen isolates with complete genomic sequence reported, showed that the degree of similarity differs across the genome, and that the 5' terminal segment is the most variable region of the PVY genome as previously reported [12]. In the nucleotide sequence analyses we found more variability in the 5'NTR than in the P1 gene (58 to 100% and 63 to 100%, respectively).

According to the antigenic and pathogenic properties showed by five PVY Mexican isolates (Pic1, Pic3, Vic6, Vic15 and Vic20) the serotype and the pathotype of three necrotic isolates, Pic1, Vic6 and Vic15, they agreed. Different results were obtained with Pic3 and Vic20 isolates wich might belong to pathotype "N" and serotype "O" (Table 1). Isolates with similar traits to Pic3 and Vic20 isolates have been reported in Poland, Canada, Spain and France [3,15,17,19,20]. For instance, the PVY^N^W (Wilga) isolate discovered in Poland in 1984, was first described as differing in virulence and aggressiveness in potato from the earlier PVY^N ^isolates and was later shown to be serologically related to PVY^O ^isolates [14]. Additionally, the nucleotide sequence comparisons from CP gene of Pic3 and Vic20 isolates (pathotype "N" and serotype "O" both isolates) with seventy isolates from outside Mexico, showed a 99% similarity with PVY^O ^isolates and clustered with other fifteen PVY^O ^isolates analyzed (data not shown).

Despite the high variability of the 5'NTR, the clustering of the twenty-nine isolates here analyzed can be arranged in two groups, "O-N" and "N-NTN" (data not shown). The PVY^O^-Pic3 Mexican isolate grouped with PVY^N^-Wi-P (from Poland) and the PVY^O^-Vic20 grouped with PVY^O^-PO7 (from Canada). The sequences of Mexico and Poland isolates showed a sequence similarity of 99%, while the sequences from other subcluster (Mexico-Canada) showed a similarity of 96%. This specific and apparently discordant clustering of two PVY^O ^Mexican isolates with isolates from different geographic regions is similar to the one observed in other works [15,21]. Two Canadian isolates (I-136 and I-L56) were found to be closely related to the PVY^N ^N242 (European) isolate in the 5'NTR region with 99% nucleotide similarity [15].

The dendrogram obtained using thirtyfour P1 gene whole sequences (data not shown) shows two main groups, the O-N and the N-NTN groups. The three Mexican isolates of the N strain cluster with seven European isolates in "N-NTN" group but in different subgroup of nine North American isolates. Interesting is the cluster "O-N" grouping three isolates: two from Mexico and one from Poland, which have the highest similarity values among the thirtyfour sequences analyzed: 96.5% (between Vic20 and Wi-P) and 98.9% (between Pic3 and Wi-P). This subgroup of new isolates is located in an independent branch different from the "N-NTN" and "O-N"groups (data not shown).

There is growing evidence that RNA recombination is a major evolutionary factor in plant RNA viruses. In our study, from the four crossover areas detected between PVY^O^-like and PVY^N^-like sequences, one putative recombination site was found in the P1 gene of several PVY^N^W isolates. Moreover, in the P1 N-terminal region (at position 499–500) of the PVY^N^Wi-P isolate there seems to be a switching from PVY^O^- to PVY^N^-like sequence [15,16].

In this work with thirty four P1 gene sequences were analyzed and potential crossover recombination sites were detected in the nucleotides 297, 301, 321 and 825 (Figure 1), resulting from a possible recombination event between the N 5yt isolate (from North America and potential parent) with the Pic3 and Vic20 isolates. This result led us to analyze the same thirty four P1 gene sequences (each one of 825 nucleotides) in two regions: the first, from 3' terminal region of 519 nucleotides and, the second, from 5' terminal region of 306 nucleotides. Two different clusters for both PVY^O ^Mexican isolates (Pic3 and Vic20) were noticed. Firstly, the analysis of thirtyfour sequences of the 3' terminal region of the P1 gene (519 nucleotides) clustered both PVY^O ^Mexican isolates within group of PVY^N-NTN ^isolates (data not shown). On the other hand, the analysis of the 5' terminal region (306 nucleotides) produced a clustering of PVY^O ^Mexican isolates within the group of PVY^O-N ^isolates (Figure 2).

## Conclusion

This is the first report of PVY necrotic strains in Mexico with two PVY isolates belonging to O strain (by serologic detection) that could be described as PVY^N^-like isolates (Wilga strains). Based in our analysis and observations, we suggest that the virus determinants of tobacco necrosis may be localized at the 3' end of the PVY P1 gene.

## Methods

### Viral isolates and bioassays

Five isolates of PVY were collected from potato plants in the State of Mexico. The infected plants in all cases showed distinct mottling and leaf distortion. The plants were maintained the greenhouses of the Dirección General de Sanidad Vegetal (SAGARPA, Mexico) during the course of this study.

The PVY isolates were inoculated in six host species maintained at greenhouse conditions on natural daylight periods (about 12 hours) and temperatures ranging between 15° to 26°C. *Chenopodium quinoa, Capsicum annuum *(var. Serrano),*Solanum lycopersicum, Nicotiana tabacum *(cv. Burley), *Physalis floridana *and *Solanum tuberosum *(cv. Alpha) were the host species used, and ten plants from each specie were inoculated with each one of the Mexican isolates.

### ELISA testing

Serological tests were performed by double-antibody sandwich enzyme-linked-immunosorbent assay (DAS-ELISA) for detection of PVA, PLRV, PVS, PVX and PVY, using commercial buffers and antibodies from AGDIA (catalogue number in parenthesis). The tests were carried out with: PAbs for PVA (SRA-60000), PLRV (SRA-30002), PVS (SRA-40000), PVX (SRA-10000), PVY (SRA-20001) and Mabs for PVY^O+C ^(SRA-20600), PVY^C ^(SRA-20700), PVY^N ^(SRA-26000). A positive result was taken as an absorbance (at 405 nm) of three times the mean of the corresponding negative control after incubation for 1 h at room temperature

### RT-PCR and sequencing

RNA was extracted from the same plants used for ELISA test using the TRIZOL^® ^method (Gibco BRL). DNA amplification of the 5'NTR and P1 regions was done in two steps: the first one to perform the reverse transcription (RT) reaction and later the PCR. RT step was done using the enzyme M-MLV RT (Promega Corporation. Madison, WI, USA), using the reverse primer 3P1R (5'-AGGATATCTCATTCGTGCCC-3') in order to reverse transcribe the 5'NTR-P1 genomic region. The same primer used in RT and the forward primer G-121 (5'-AATTAAAACAACTCAATACAACATAAGAAA-3') were used in the PCRs. The amplified products were cloned in the pGEM-T Easy vector (Promega Corporation, Madison, WI, USA). Nucleotide sequencing of cloned PCR products was carried out on plasmid minipreps (High Pure Plasmid Isolation Kit, Hoffman-La Roche, LTD, Basel, Switzerland)) using an Abi Prism 377 Perkin-Elmer automated sequencer (Cetus, Norwalk, CT).

### Sequence analysis

In order to carry out a detailed comparison of the 5'NTR region and the P1 gene of PVY, sequence alignments were made of 29 and 34 different isolates, for 5'NTR region and P1 gene respectively. The 5'NTR region analysis included 3 PVY^O ^and 21 PVY^N/NTN ^isolates obtained from the National Center for Biotechnology Information (Table 2), along with sequences from the two PVY^O ^and three PVY^N ^isolates described in this paper (Table 1). The P1 gene analysis included 6 PVY^O ^and 23 PVY^N/NTN ^isolates obtained from sequence databases (NCBI) (Table 2), along with the two PVY^O ^and three PVY^N ^sequences isolated in Mexico (Table 1). The sequence analysis was made using the whole sequence of 5'NTR or P1 gene.

**Table 2:** Thirtyone PVY sequences from database of NCBI used in the comparisons and analysis of 5'NTR and P1 gene sequences

**ACCESSION NUMBER**	**ISOLATE**	**PVY strain**	**COUNTRY**
AF237963	PVY-pvn	N	ITALY
AF248499	PVYN-N242	N	FRANCE
AF248500	PVYN-Wi-P	N	POLAND
AF401600	N 266	N	North America
AF401601	N 394	N	North America
AF401602	S1 44	NTN	SLOVENIA
AF401603	S1 50	NTN	SLOVENIA
AF401604	S1 64	NTN	SLOVENIA
AF401605	N 5yt	N	North America
AF401606	N 27	N	North America
AF401607	N Jg	N	North America
AF401608	Tu 619	NTN	North America
AF401609	Tu 660	NTN	North America
AF401610	Tu 648	NTN	EUROPE
AF522296	N-Egypt	N	EGYPT
AJ245554	Loimaa	O	FINLAND
AJ245555	803	O	FINLAND
AJ245556	Viikki	O	FINLAND
AJ245557	RUS	N	RUSSIA
AJ245558	UK	N	U. K.
AY166866	Tu 660	NTN	CANADA
AY166867	N-Jg	N	CANADA
AY178846	PVY-O	O	INDIA
D00441	Fr	N	FRANCE
M37180	na	O	Na
M38377	na	C	Na
M95491	Hungarian	NTN	HUNGARY
U09509	PO7	O	CANADA
X12456	Fr	N	FRANCE
X82848	na	O	FINLAND
X97895	605	N	SWITZERLAND

na: data unavailable

Multiple alignment of the 5'NTR and P1 genes of nucleotide sequences (and amino acid sequences for P1 gene) were obtained using the CLUSTALX, DNAStar and PAUP package. Phylogenetic relationships were determined in the MEGA3 package. Distance matrices were calculated with the Kimura two-parameter option, and distance trees were constructed from these matrices by the Neighbor Joining method. Other methods tested like UPMGA, Minimum Evolution and Maximum Parsimony gave similar results, with little significant difference. A value for each internal node was estimated for statistical significance of branching by performing 10,000 replications of the bootstrap resampling from the original data. For recombination analysis in the 5'NTR and the P1 gene the Recombination Detection Program (RDP) was used. Pairwise identity plots were used to identify possible recombinant regions.

## Competing interests

The authors declare that they have no competing interests.

## Authors' contributions

VRRR carried out the molecular genetic studies, participated in the sequence alignments and prepared tables and figures. KAP participated in the sequence alignments and partially drafted the final manuscript. GFT collected viral samples and organized information and material data. LSR participated in the design of the study and performed some of the statistical analysis. JPMS conceived of the study, and participated in its design and coordination. All authors read and approved the final manuscript.
