# Presence of necrotic strains of *Potato virus Y *in Mexican potatoes

**DOI:** 10.1186/1743-422X-6-80

**Published:** 2009-06-18

**Authors:** Victoriano Roberto Ramírez-Rodríguez, Gustavo Frías-Treviño, Katia Aviña-Padilla, Juan Pablo Martínez-Soriano

**Affiliations:** 1 Centro de Investigación y de Estudios Avanzados del Instituto Politécnico Nacional, Campus Guanajuato, km. 9.6 libramiento norte, carretera Irapuato-León, 36821 Irapuato, Guanajuato, Mexico; 2 Universidad Autónoma Agraria Antonio Narro, Departamento de Parasitología. Buenavista, Saltillo, Coahuila, Mexico

## Abstract

Correction to Ramírez-Rodríguez VR, Frías-Treviño G, Aviña-Padilla K, Silva-Rosales L, Martínez-Soria JP: Presence of necrotic strains of Potato virus Y in Mexican potatoes. Virology Journal 2009, 6:48

## Correction

The published version of this article [1] included Dr Silva-Rosales in the author list. Dr Silva-Rosales was not involved in the detailed process of this manuscript, and has requested to be removed from the author list. Therefore, we are removing Dr Silva-Rosales as an author; a corrected author list has been provided with this Correction.
